# The Stiffness for Viscous Deformation in the Interlamellar Amorphous Region of Polyethylene

**DOI:** 10.3390/polym18010130

**Published:** 2025-12-31

**Authors:** P.-Y. Ben Jar, Na Tan, Salman Obaidoon, Arash Alizadeh, João B. P. Soares

**Affiliations:** 1 Department of Mechanical Engineering, University of Alberta, Edmonton, AB T6G 1H9, Canada; ntan2@ualberta.ca; 2 Department of Chemical & Materials Engineering, University of Alberta, Edmonton, AB T6G 1H9, Canada; obaidoon@ualberta.ca (S.O.); arash.alizadeh@ualberta.ca (A.A.); jsoares@ualberta.ca (J.B.P.S.)

**Keywords:** MR test, spring–dashpot model, stiffness of the interlamellar region, polyethylene

## Abstract

A spring–dashpot model, consisting of a spring branch and two Maxwell (named long- and short-term) branches, was used to simulate stress drop during the relaxation stages of multi-relaxation (MR) tests. This work shows that the stress drop at relaxation in a deformation range around the peak stress could be closely simulated without changing the parameter values for the short-term branch. This possibility was confirmed using three ethylene/1-hexene copolymers and one ethylene homo-polymer, among which the main differences are mass density and short-chain branch (SCB) content. The work examined the influence of SCB content and mass density on the stiffness of the two Maxwell branches, and the results showed that, unlike the long-term branch counterpart, stiffness of the short-term branch is not a monotonic function of the SCB content or the mass density. This led to a discussion on the possible relationship between the stiffness of the two Maxwell branches and the deformation resistance of the amorphous phase at different locations of the microstructure, i.e., in the interlamellar region and as part of the network structure. The paper concludes that a combination of the MR test and the spring–dashpot model could provide information that is related to the stiffness in different parts of PE’s amorphous phase, though further work is needed to verify this conclusion.

## 1. Introduction

Spring–dashpot models have long been applied to polymers for simulating mechanical test results. These models have also been used to analyze viscous stress responses to deformation, which, after calibration, can indeed closely mimic empirical data, but may not provide a meaningful interpretation, especially for relating stress variation to microstructural changes. Consequently, even though these models have long been used to simulate viscous deformation behavior [1,2,3,4], they have rarely been used to provide physical meanings of mechanical properties. The overall objective of our study is to examine the possibility of using spring–dashpot models to characterize mechanical properties for semi-crystalline polymers that consist of crystalline, constrained amorphous, and mobile amorphous phases [5,6]. This paper uses polyethylene (PE) as the sample material.

PE was selected for the study because it consists of amorphous and crystalline phases [7,8], with the former being located either between crystalline lamellae or as part of the network structure that connects lamella clusters [9,10]. The amorphous phase between the crystalline lamellae appears as thin layers that, compared to the amorphous phase in the network structure, have relatively strong resistance to deformation due to the constraints from the adjacent crystalline lamellae on transverse deformation. The thicker the lamellae are, the stronger the constraints provided are. This leads to an increase in the stiffness of the interlamellar region. However, stiffness of the interlamellar region may also increase by an increase in inter-molecular friction during disentanglement, which can be achieved by introducing short-chain branches (SCBs) on PE molecules [11]. Since an increase in SCB content may also decrease lamellar thickness and, thus, reduce its constraint on the transverse deformation of the interlamellar region [12], it is not always clear what the net effect of the SCB content would be on the stiffness of the interlamellar region.

The above issue on the stiffness enhancement of the interlamellar region has been considered by many research groups in the last four decades, and the literature has been thoroughly reviewed by Guo et al. [13], who further investigated the stiffness difference in the amorphous phase in terms of the apparent amorphous modulus before yielding and after extensive necking. Our work has similar interests, but focuses mainly on deformation before extensive necking, with time as an independent variable in order to investigate viscous deformation behavior.

The work described here is part of the effort to establish the relationship between long-term mechanical performance and short-term laboratory test results. This paper describes a methodology that uses a spring–dashpot model to measure viscous stiffness of four PEs with different SCB contents and mass densities, based on which a discussion is made on the feasibility of using this methodology to evaluate the influence of SCB content and mass density on the stiffness of the interlamellar region.

## 2. Methods for Material Characterization, Mechanical Testing, and Modeling

### 2.1. Material Characterization

Three ethylene/1-hexene copolymers and one ethylene homo-polymer, with a Ziegler–Natta catalyst—supplied as compression-molded plaques with a nominal thickness of 3 mm—were used in this study. The plaques were fabricated under molding conditions that were used to generate the product data sheets for these PE resins. The supplier of these materials (which requested to remain anonymous) used ASTM D1505 [14] and ASTM D638 [15] to determine mass densities and tensile yield strengths, respectively, for these plaques.

In addition to the supplier-provided mass densities and tensile yield strengths, these PE samples were also characterized using a Polymer Char high-temperature gel permeation chromatograph (GPC) operated at 145 °C and calibrated with narrow polystyrene standards. For the GPC analyses, the PE samples were prepared as dilute solutions in distilled trichlorobenzene (TCB) and injected into a continuously flowing TCB stream at a flow rate of 1 mL/min. The solutions passed through three columns packed with cross-linked polydivinyl benzene particles (Agilent PLgel Olexis, Santa Clara, CA, USA, 7.5 × 300 mm, 13 µm particles). The GPC analyses measured the weight- and number-average molecular weights (*M_w_* and *M_n_*, respectively), polydispersity index (PDI), and SCB content (using an online infrared detector), expressed as the number of short-chain branches per 1000 backbone carbons (SCBs/1000 C) of the PE samples.

Table 1 lists the properties of these PE samples, showing that the tensile yield strength increases with the density. The table also shows that the most significant differences among the four PE samples are their SCB contents and mass densities, with the former ranging from 6.4 SCBs/1000 C for PE #1 to 0 for PE #4. Although the molecular weight averages for PE #3 and PE #4 are slightly lower than those for PE #1 and PE #2, the decrease in the molecular weight was not high enough to affect the trend of increase in the yield strength with density, and as suggested in Ref. [11], the decrease in SCB content could also contribute to the slightly lower molecular weight.

### 2.2. Multi-Relaxation Test and Specimen Geometry

Multi-relaxation (MR) test is a mechanical test method that introduces multiple stages of loading and relaxation to one specimen. The loading is under stroke control, at a crosshead speed of 5 mm/min, and the relaxation is at constant specimen elongation for a period of 10,000 s. The MR tests conducted herein were based on a test program similar to that used in a previous study [16]. That is, a displacement increment of about 0.224 mm was introduced at each of the loading stages at a crosshead speed of 5 mm/min. Before the yield point, this displacement increment corresponded to an areal strain increment of about 0.7%, with the areal strain being defined as $2\times ln\left(W_{o}/W_{d}\right)$, where $W_{o}$ is the original width in the gauge section of the specimen and $W_{d}$ the width of the deformed gauge section at the same location. The main modification of the test program used herein was to increase the number of data points collected at the beginning of the relaxation stages so that a sufficient number of points were available for determining the time for the onset of relaxation. These additional data points were found to be essential for closely fitting values generated from a three-branch spring–dashpot model at the beginning part of the stress drop curve at the relaxation stages. Details for determining the onset point for the relaxation were given in a previous publication [17].

It should be noted that the MR test used in this study is similar to the cyclic step relaxation test reported in the literature [13]. Both test methods introduce small—but sufficient—amounts of deformation at the loading stage so that, before the yield point, the stress applied at the end of a loading stage is always higher than the corresponding stress at the previous loading stage. This is to ensure that additional microstructural changes could be introduced at each loading stage.

All MR tests were stopped either when fracture occurred (for PE #4) or at a stroke of about 6 mm, where the applied stress had passed the peak point and reached the plateau region. Based on the reduction in the cross section in the gauge section, the deformation introduced at a stroke of about 6 mm is equivalent to an areal strain of about 0.4, at which point neck formation has started but has not reached its final dimensions. Duplicated MR tests were conducted to ensure good reproducibility of the test results.

**Figure 1:**
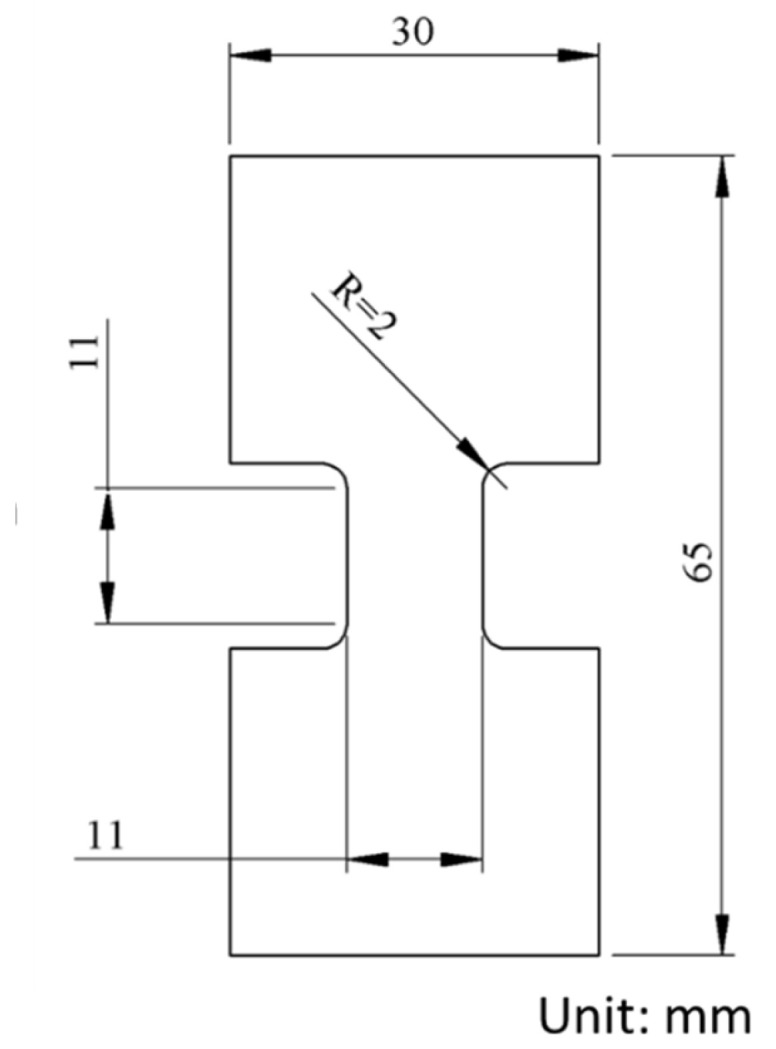
Specimen dimensions used for the MR tests.

Dog-bone specimens used for the MR test were water-jetted directly from the compression-molded plaques, with the in-plane dimensions shown in Figure 1. The MR tests were conducted at room temperature using a universal test machine (Qualitest Quasar 100, by GALDABINI in Cardano al Campo, Italy), with the test program and data acquisition controlled by a personal computer.

### 2.3. Spring–Dashpot Modeling and Data Analysis

**Figure 2:**
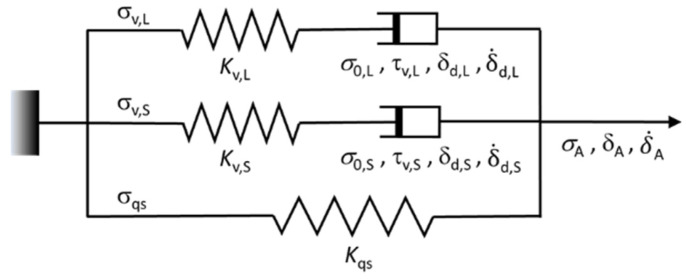
The three-branch spring–dashpot model used in this study.

Several spring–dashpot models have been used in the literature to simulate PE’s mechanical test results for several purposes. For example, a three-branch model that consists of one Maxwell branch connected in parallel with one spring branch and one spring–plastic element branch was used to simulate results from a ‘step-cycle’ tensile test of a poly(ethylene-co-vinyl acetate) sample [18]. The main objective of that study was to separate the quasi-static stress response of the network structure from the stress response of the crystallites, without paying much attention to the reproduction of stress drop at the beginning of relaxation, when significant viscous stress contributes to the stress drop. Another work, also from Strobl’s group [19], used a two-branch model, i.e., one Maxwell branch and one spring–plasticity element branch, to fit the stress drop at the beginning of relaxation, but the fitting was performed only up to a relaxation time of 3000 s, while the entire relaxation period used in the study was 10,000 s. Sweeney et al. [2] suggested that rather than a spring–dashpot model with only one Maxwell branch, a model with at least two Maxwell branches would be needed to provide a reasonable fitting of the stress response of a shape memory polymer at relaxation and recovery after loading and unloading, respectively. Shi and Jar [20] also showed that a parallel three-branch model with two Maxwell branches could provide a close simulation of stress drop during relaxation and stress increase during recovery, each for a period of 10,000 s. However, not all spring–dashpot models that contain two dashpots could have such a capability, especially for mimicking the stress decrease at a later stage of stress recovery [21]. Based on the above works, the current study adopted a parallel three-branch spring–dashpot model with two Maxwell branches (Figure 2), which was the same model as that used in a previous study [17], to mimic the entire stress drop curves at all relaxation stages of the MR test.

As shown in Figure 2, the bottom branch (with subscript ‘qs’) contains only a spring; it is used to mimic the quasi-static stress response. The middle and top branches are Maxwell branches, which are used to model the stress drop at the relaxation stages. These two Maxwell branches were named short- and long-term branches (with subscripts ‘S’ and ‘L’, respectively), based on the magnitude of their characteristic relaxation time ($\tau_{v,S}$ and $\tau_{v,L}$, respectively).

A previous study [17] has shown that the stress drop obtained at relaxation stages, $\Delta \sigma_{A}\left(t\right)$, could be regenerated using the three-branch model by determining values for the following parameters: viscous stress at the beginning of the relaxation stage ($\sigma_{v,i}\left(0\right)$), reference stress ($\sigma_{0,i}$), and characteristic relaxation time ($\tau_{v,i}$), where i = S for the short-term branch and i = L for the long-term branch. The expression for $\Delta \sigma_{A}\left(t\right)$ as a function of these parameters is given below, for which the derivation has been provided in the literature [17,22,23]:

(1) $$\begin{array}{ll} \Delta \sigma_{A}\left(t\right)\; & =\sigma_{A}\left(0\right)\;-\sigma_{A}\left(t\right)\;=\Delta \sigma_{v}\left(t\right)\\ & =\sigma_{v,L}\left(0\right)\;-\;2\sigma_{0,L}{tanh}^{-1}\left\{tanh\left[\sigma_{v,L}\left(0\right)/\left(2\sigma_{0,L}\right)\right]\;exp\left(-t/\tau_{v,L}\right)\right\}\\ & +\sigma_{v,S}\left(0\right)\;-\;2\sigma_{0,S}{tanh}^{-1}\left\{tanh\left[\sigma_{v,S}\left(0\right)/\left(2\sigma_{0,S}\right)\right]\;exp\left(-t/\tau_{v,S}\right)\right\} \end{array}$$

where $\sigma_{A}$ is the applied stress at each relaxation stage, and $\Delta \sigma_{v}$ is the corresponding viscous stress drop, both of which are functions of time, *t*, measured from the beginning of a given relaxation stage.

After the values were determined for the above fitting parameters, quasi-static stress ($\sigma_{qs}$) was then determined as a function of stroke using the following expression:

(2) $$\sigma_{qs}=\sigma_{A}\left(0\right)\;-\;\sigma_{v,L}\left(0\right)\;-\;\sigma_{v,S}\left(0\right)$$

**Figure 3:**
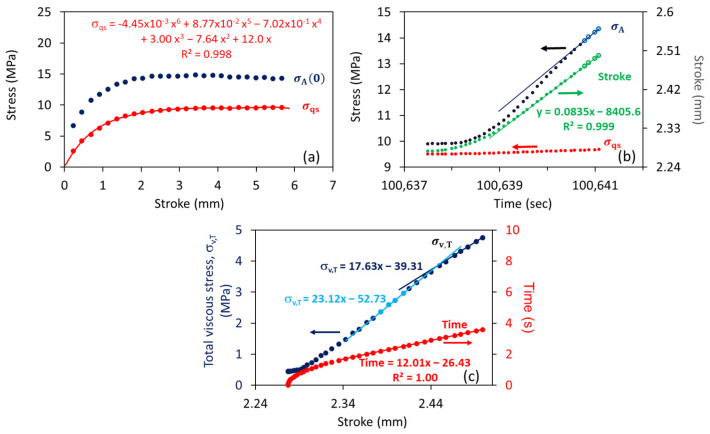
An example of plots established from the testing and the modeling: (**a**) $\sigma_{A}\left(0\right)$ and $\sigma_{qs}$ as functions of stroke; (**b**) $\sigma_{A}$, $\sigma_{qs}$, and stroke at a loading stage as functions of test time; and (**c**) $\sigma_{v,T}$ and time from the beginning of the loading stage as functions of stroke.

Figure 3 presents an example of the data analysis used in this study. Figure 3a shows a typical variation in $\sigma_{qs}$ with an increase in the stroke, along with its fitting curve based on a 6th-order polynomial function, and the corresponding value for the applied stress at the beginning of the relaxation stages, $\sigma_{A}\left(0\right)$. Figure 3b shows an example of the variation in applied stress ($\sigma_{A}$), stroke, and $\sigma_{qs}$ at a given loading stage as functions of test time, in which the values for $\sigma_{qs}$ were calculated using the polynomial function given in Figure 3a. Figure 3b indicates that after the stroke rate reached the programmed speed of 5 mm/min (i.e., 0.0835 mm/s, which was reached at a test time of 100,639 s in the figure), the rate of increase in $\sigma_{A}$ at the loading stage showed a transition that was similar to the transition observed before [17], which has been suggested to be caused by the dashpot in the short-term branch reaching the programmed speed of 5 mm/min and, thus, no longer contributing to the increase in $\sigma_{A}$ with the increase in stroke. A similar transition is shown in Figure 3c for the total viscous stress ($\sigma_{v,T}$), which is equivalent to the difference between $\sigma_{A}$ and $\sigma_{qs}$ in Figure 3b, though, in Figure 3c, $\sigma_{v,T}$ is expressed as a function of stroke, rather than time. Therefore, the slope of the $\sigma_{v,T}$ curve in Figure 3c at the end of the loading stage (17.63 MPa/mm) represents the stiffness of the long-term branch, while the slope before the transition (23.12 MPa/mm) is the total stiffness of the two viscous branches. And the difference between the two slopes is the stiffness of the short-term branch (5.49 MPa/mm).

In view that the use of the above approach to simulate the MR test results could yield multiple sets of parameter values [20], the current study considered one additional condition for the data analysis, that is, values for the parameters in the short-term branch ($\sigma_{v,S}\left(0\right)$, $\sigma_{0,S}$, and $\tau_{v,S}$) did not change in the stroke range from 1.5 to 4.3 mm, corresponding to an areal strain range from 0.065 to 0.27. Considering that the critical strain for fibrillation of the crystalline lamellae is around 0.6 [22], the crystalline lamellae should remain largely undisturbed in the above stroke range of the MR tests.

The criterion for the use of the spring–dashpot model to fit the stress drop curves at the relaxation stages of the MR tests was that the difference between the experimentally measured stress drop value and that obtained from the three-branch model should be no more than 0.08 MPa. If this fitting criterion could not be satisfied in the stroke range from 1.5 to 4.3 mm, the fitting process would start again using a different set of fitting parameter values for the short-term branch.

It should be noted that the three-branch model used in this study did not include a plasticity element in the quasi-static branch, which is different from the models used by Strobl’s group [18,19,22,23]. This is because the focus of our study was on PE’s stiffness for viscous deformation, which would not be affected by removing the plasticity element in the quasi-static branch and, thus, simplifies the data analysis. Moreover, the analysis conducted on the MR test results was focused on deformation up to the yield point, at which the plastic deformation generated in the specimens should be negligible.

## 3. Test Results and Stiffness Calculation

### 3.1. Test Results

**Figure 4:**
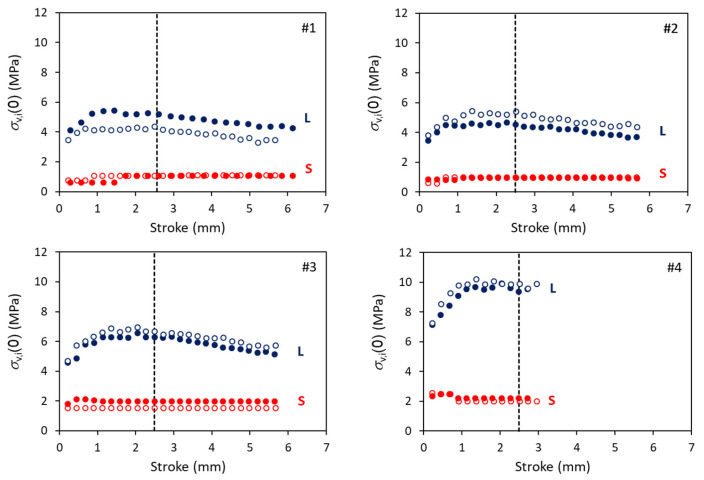
Summary of $\sigma_{v,i}\left(0\right)$ values (i = S and L) determined for the long- and the short-term branches (labelled with L and S, respectively). Solid and hollow markers in each plot are from different specimens.

Figure 4 summarizes values for $\sigma_{v,i}\left(0\right)$ (i = S and L) for short- and long-term branches, respectively, for mimicking the stress drop curves at the relaxation stages of the MR tests. The corresponding values for $\sigma_{0,i}$ and $\tau_{v,i}$ are given in Appendix A. A vertical dashed line is used to mark a stroke of 2.5 mm, around which the applied stress, $\sigma_{A}\left(0\right)$, reached a plateau, as shown later. Figure 4 suggests that the $\sigma_{v,S}\left(0\right)$ values are always smaller than the corresponding $\sigma_{v,L}\left(0\right)$, and that the former could remain constant in the stroke range from 1.5 to 4.3 mm. The phenomenon of constant value is also shown for $\sigma_{0,S}$ and $\tau_{v,S}$, as depicted in Appendix A.

**Figure 5:**
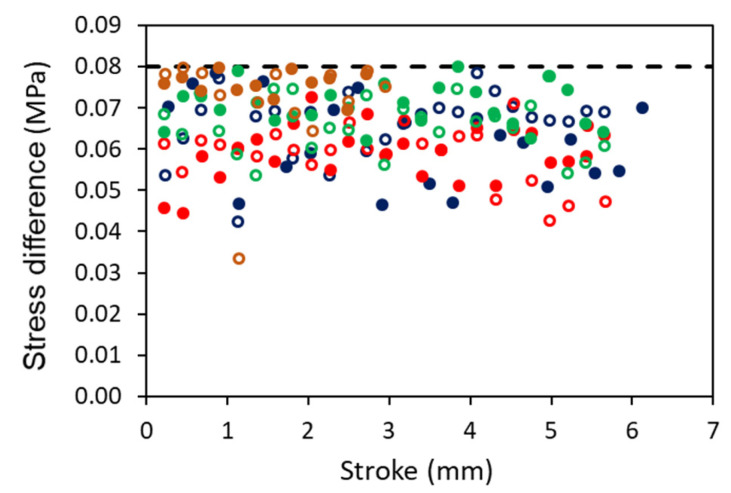
Summary of the maximum difference between values generated from the three-branch model and the experimental measurements at all relaxation stages in the MR tests conducted in this study. Different colors are for different specimens.

The maximum differences between the curve of stress drop generated from the three-branch model and that from the experimental measurements at a given stroke are summarized in Figure 5. The figure indicates that the fitting errors were no more than 0.08 MPa, which is much smaller than those reported in the literature [2,16,24,25,26].

**Figure 6:**
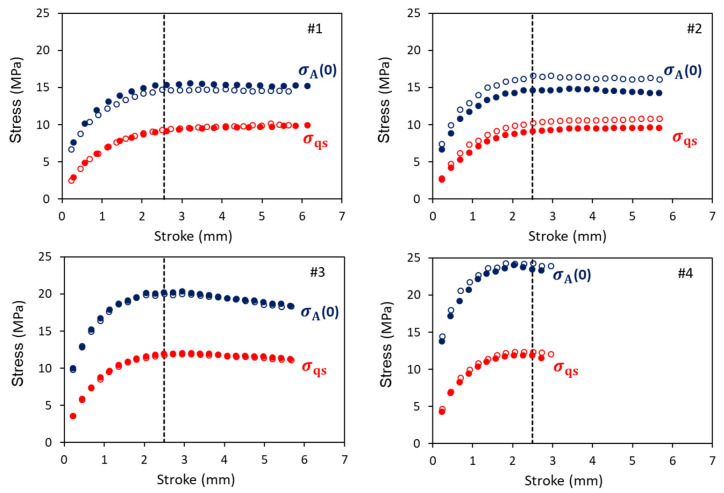
Summary of $\sigma_{A}\left(0\right)$ and $\sigma_{qs}$ as functions of stroke for the 4 PEs. Solid and hollow marks are from different specimens.

Figure 6 summarizes the $\sigma_{qs}$ and $\sigma_{A}\left(0\right)$ values, each expressed as a function of stroke. Similar to that in Figure 4, a vertical dashed line is used to mark a stroke of 2.5 mm, which is the stroke for $\sigma_{A}\left(0\right)$ of PEs #1, #2, and #3 to reach a plateau. For PE #4, the plateau is not as obvious and occurs at a stroke slightly smaller than 2.5 mm. This is possibly because specimens of PE #4 fractured at a stroke of around 3 mm, and thus, at a stroke of 2.5 mm, damage could have been generated, resulting in a stress drop. If the damage had been prevented, as suggested in the literature [27,28], the stroke at the peak stress could have been larger. For convenience and consistency, a comparison of the simulation results presented herein among the four PEs is based on data obtained from the relaxation stage at a stroke that is closest to 2.5 mm.

**Figure 7:**
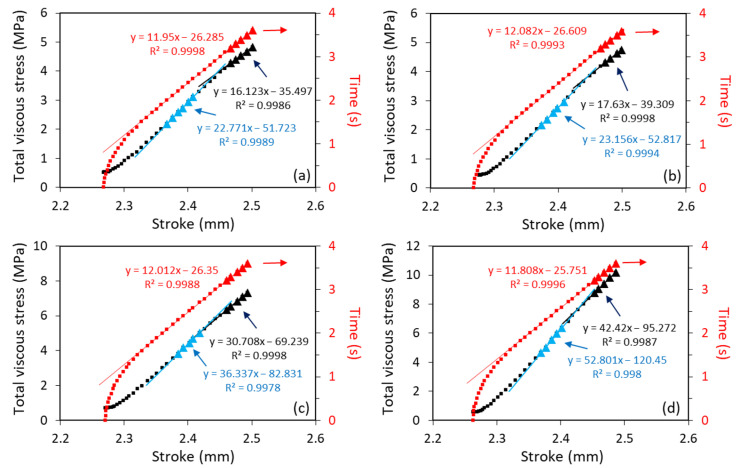
Sample curves of total viscous stress and test time as functions of stroke at the loading stage prior to relaxation at a stroke around 2.5 mm.

Figure 7 presents sample curves of total viscous stress, i.e., the summation of $\sigma_{v,L}\left(0\right)$ and $\sigma_{v,S}\left(0\right)$, and test time as functions of stroke at a loading stage prior to relaxation at the stroke closest to 2.5 mm. The figure shows that after the rate of stroke increase reaches 5 mm/min (i.e., in Figure 7 at the stroke above 2.3 mm), the curves of total viscous stress show a bi-linear profile, similar to that observed in a previous study [17]. As suggested before, this bi-linear curve profile could be caused by the constant stress contribution from the short-term branch in the second part of the loading stage. Using the analysis depicted in Figure 3, the values for the two slopes at the loading stage prior to relaxation at a stroke around 2.5 mm are summarized in Table 2.

### 3.2. Stiffness Calculation

**Figure 8:**
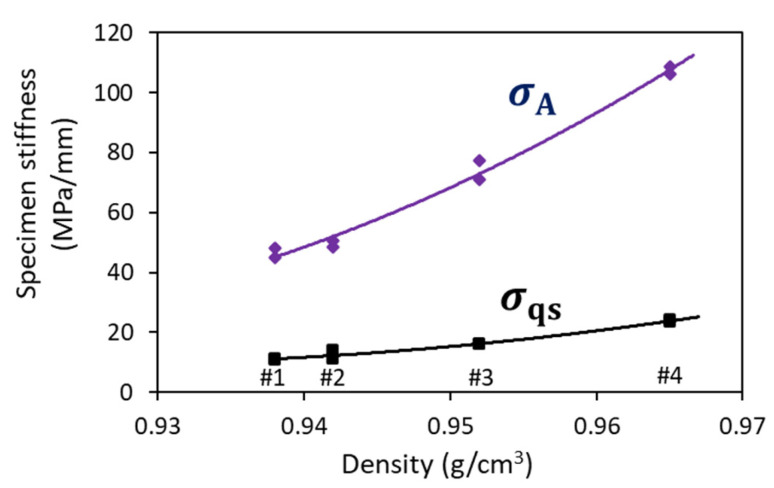
Stiffness values of PE specimens and stiffness trend lines based on the initial slope of $\sigma_{A}$–stroke and $\sigma_{qs}$–stroke plots from the MR tests.

Traditional calculation for the overall stiffness of a polymer specimen is based on the initial slope of the applied stress–displacement curve. In this study, because of the possibility of extracting quasi-static stress from the applied stress, specimen stiffness could be determined based on either the applied stress $\left(\sigma_{A}\right)$ or the $\sigma_{qs}$ value, which are summarized in Figure 8 as a function of mass density. Since the difference between the two stiffness values in Figure 8 represents the contribution of viscous stress to the overall stiffness of the specimen, the figure suggests that viscous stress has played a dominant role in the overall stiffness of the four PEs, and that these stiffness values increase monotonically with an increase in mass density. Such a trend of stiffness increase in Figure 8 is consistent with works on elastic moduli reported in the literature [9,29,30].

**Figure 9:**
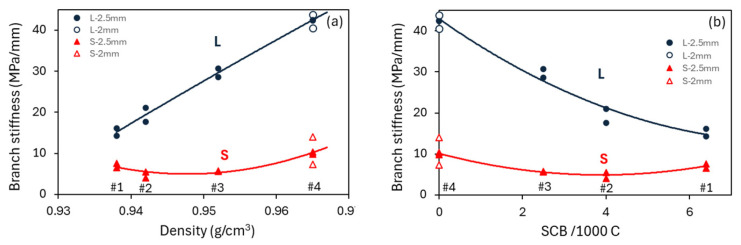
Summary of the branch stiffness for the long- and short-term branches, marked by L and S, respectively, as functions of PE’s mass density (**a**) and SCB content (**b**). The filled marks are for data at a stroke of 2.5 mm, and the unfilled marks for PE #4 at a stroke of 2 mm.

As discussed in Section 2.3 and depicted in Figure 3c, we have attributed the increase in the second part of the $\sigma_{v,T}$–stroke plot, as shown in Figure 7, to the stress increase in the long-term branch of the spring–dashpot model. Therefore, the corresponding slope for the second part of the curve in Table 2 represents the stiffness of the long-term branch. This also suggests that the difference in the slopes between the two parts of the $\sigma_{v,T}$–stroke plot at the loading stages, such as those listed in Table 2 at a stroke of 2.5 mm, represents the stiffness of the short-term branch. These stiffness values at a stroke of 2.5 mm are summarized in Figure 9, using filled marks, with L and S indicating the long- and short-term branches, respectively. Figure 9 also includes unfilled marks for PE #4, which represent the branch stiffness for PE #4 at a stroke of 2 mm, where, as shown in Figure 6 for PE #4, both $\sigma_{A}\left(0\right)$ and $\sigma_{qs}$ reached the peak value. Figure 9a presents the variation in the branch stiffness as a function of mass density, and Figure 9b as a function of SCB content. For the long-term branch, the two figures suggest that its stiffness increases monotonically with an increase in mass density and a decrease in the SCB content, which shows a trend consistent with the trend of modulus change reported in the literature [29,30]. For the short-term branch, however, variation in its stiffness does not show a monotonic change with either the mass density or the SCB content. Rather, data for PE #3 and #4 show an increase in branch stiffness with an increase in mass density and a decrease in SCB content. On the other hand, for PE #1 and #2, the branch stiffness increases with decreases in mass density and an increase in SCB content, i.e., in an opposite way to that for PE #3 and #4.

### 3.3. Discussion

Results from some previous studies [20,31] have shown that the use of a spring–dashpot model with the stress response of the dashpot governed by Eyring’s Law [32] could generate multiple sets of values for the model parameters, all of which enabled the spring–dashpot model to regenerate the stress drop at the relaxation stages of the MR test. In the current study, we chose a particular set of parameter values so that the values for the parameters in the short-term branch remained constant around the peak stress, that is, in a stroke range from 1.5 to 4.3 mm. This choice of parameter values was selected to generate a cyclic deformation mode for the spring in the short-term branch, that is, to allow the stretch of the spring to reach the same limit before the end of a loading stage in the above stroke range, resulting in the same steady-stress contribution from the short-term branch in the remaining part of the loading stages in that stroke range. This was conducted to mimic the deformation behavior of PE’s interlamellar region in the above stroke range of the MR test, as the integrity of the crystalline lamellae below a stroke of 4.3 mm (i.e., above an areal strain of 0.27) should remain relatively undisturbed. However, below a stroke of 1.5 mm (i.e., with an areal strain lower than 0.067), there could be some stretch introduced to the interlamellar region to tauten the molecular segments.

Based on the above deformation analogy between the short-term branch of the spring–dashpot model and the interlamellar region of PE, the difference in the stiffness of the interlamellar region among the four PEs could be represented using the stiffness of the short-term branch shown at the loading stage of the MR test. Figure 9 suggests that both mass density and SCB content could affect the stiffness in PE’s interlamellar region, but in an opposite way.

Although a linear relationship has long been accepted for PE’s lamellar thickness and mass density [33], the influence of SCB content on the mass density and the lamellar thickness is yet to be fully clear [12,34,35]. Nevertheless, based on the work in Ref. [12], the SCB content is expected to follow a non-linear, inverse relationship with PE’s lamella thickness, that is, a non-linear, accelerating increase in lamellar thickness with a decrease in SCB content. For PE #3 and #4, by decreasing the SCB content from 2.5 to 0 per 1000 C, i.e., from PE #3 to #4 in Figure 9, the lamella thickness is expected to increase significantly, and thus, the resistance to transverse deformation in the interlamellar region would also increase (i.e., an increase in stiffness). On the other hand, with the decrease in the SCB content from 6.4 to 4 per 1000 C, i.e., from PE #1 to #2, the increase in the lamella thickness could not be as significant as that from PE #3 to #4, and the role of the SCB content could dominate the stiffness of the interlamellar region. That is, with the increase in SCB content from PE #2 to #1, the increase in inter-molecular friction could cause an increase in the stiffness of the interlamellar region. Therefore, the relationship of the interlamellar stiffness with PE’s SCB content and mass density could be non-linear, suggesting that the stiffness of the short-term branch in Figure 9 could provide a plausible indication for the stiffness change in the interlamellar region among the four PEs.

Although the work in the literature has not paid much attention to the stiffness for PE’s viscous deformation, research in the past has shown that an increase in lamellar thickness could increase the yield stress [36], but not the elastic modulus [37]. And for the SCB content, work using a molecular dynamics simulation has suggested that an increase in SCB content could decrease the elastic modulus and yield strength [38], but an experimental study on the influence of the SCB length on the mechanical properties of a linear low-density polyethylene (LLDPE) film did not reach such a clear conclusion [39]. Therefore, our suggestion on the stiffness of the long-term branch, as shown in Figure 9, which represents the influence of lamellar thickness and SCB content on the stiffness of the amorphous phase in the network structure of PE, may simply add an additional piece of information for an area that is yet to be fully understood.

The above discussion suggests that, although the use of the deformation resistance of the amorphous phase at different locations of PE’s microstructure could provide a plausible explanation for the results presented in Figure 9, direct evidence is still needed to support the physical interpretation of the steady-state stress contribution from the short-term branch to $\sigma_{v,T}$ at the second part of the loading stage. This requires further investigation that includes small-angle X-ray scattering to quantify the lamellar thickness of the four PEs and electron microscopy to quantify the deformation of the network structure and the change in the orientation of the lamellar clusters in the MR test. When this manuscript is prepared, we are investigating whether the steady-state stress contribution from the short-term branch is the only scenario that could be provided by the three-branch model to mimic the stress–stroke curves at all loading stages of the MR test and, furthermore, whether the bi-linear profile of the $\sigma_{v,T}$–stroke curve, as shown in Figure 7, could be caused by a transition of the deformation mechanism involved at the loading stages. Several challenges for the above studies have been identified, and the possibility of collaborating with other research groups to overcome these challenges is being explored.

## 4. Conclusions

This work has demonstrated the possibility of combining the MR test and a three-branch spring–dashpot model to characterize PE’s stiffness for viscous deformation. Using the results for four PEs of different mass densities and SCB contents, the work shows that the stiffness of the short-term branch in the spring–dashpot model could represent the dependence of the stiffness in the interlamellar region on PE’s lamellar thickness and SCB content. This offers the possibility of quantifying the mechanical properties in the interlamellar region of PE, which is being further investigated as this manuscript is prepared.

## Figures and Tables

**Table 1:** Characterization of PEs used in this study.

#	PE Type	Density, ρ (g/cm^3^)	Yield Strength (MPa)	M_w_ (g/mol)	M_n_ (g/mol)	PDI	SCB Content (SCB/1000 C)
1	LLDPE	0.938	18	74,862	20,282	3.69	6.4
2	HDPE	0.942	19	85,868	22,209	3.87	4
3	HDPE	0.952	26	62,718	16,714	3.75	2.5
4	HDPE	0.965	30	60,247	14,597	4.13	0

**Table 2:** Summary of the slope values from two specimens for each PE at the loading stages prior to relaxation at strokes around 2.5 mm.

#	PE Type	The 1st Part of the Curve (MPa/mm)		The 2nd Part of the Curve (MPa/mm)	
1	LLDPE	21.9	22.7	14.3	16.1
2	HDPE	23.1	25.1	17.6	21.1
3	HDPE	36.3	34.3	30.7	28.6
4	HDPE	52.8	52.4	42.4	42.6

**Figure A1:**
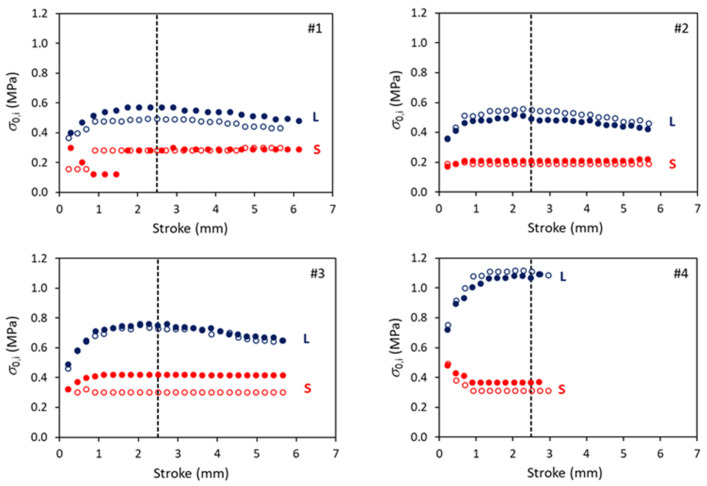
Summary of $\sigma_{0,i}$ values (i = L and S) as a function of stroke for PEs used in the study. Solid and hollow markers are from different specimens of the same type of PE, as indicated at the top, right corner in each plot.

**Figure A2:**
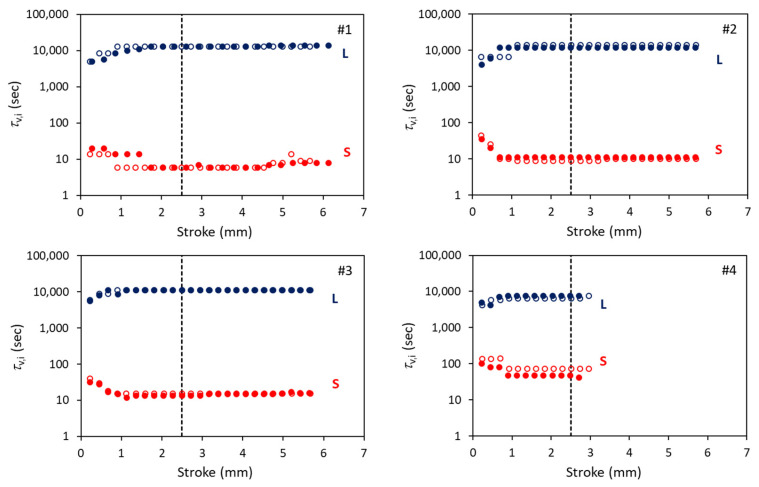
Summary of $\tau_{v,i}$ values (i = L and S) as a function of stroke for PEs used in the study. Solid and hollow markers are from different specimens of the same type of PE, as indicated at the top, right corner in each plot.

## Data Availability

The data presented in this study are available upon request from the corresponding author.

## Abbreviations

The following abbreviations are used in this manuscript:

PE	Polyethylene
SCB	Short-chain branches
GPC	Gel permeation chromatograph
TCB	Trichlorobenzene
PDI	Polydispersity index
MR	Multi-relaxation
$\tau_{v,i}$	Characteristic relaxation time in the i branch, i = S and L
$\sigma_{v,i}\left(0\right)$	Viscous stress in the i branch at the beginning of the relaxation
$\sigma_{0,i}$	Reference stress
$\sigma_{qs}$	Quasi-static stress
$\sigma_{A}(0)$	Applied stress at the beginning of the relaxation stages
$\sigma_{A}$	Applied stress
$\sigma_{v,T}$	Total viscous stress

## Appendix A

Figure A1 and Figure A2 summarize the $\sigma_{0,i}$ and $\tau_{v,i}$ values, respectively, for long- and short-term branches (i = L and S, respectively) of the three-branch spring–dashpot model to regenerate the stress drop at the relaxation stages of the MR tests. The corresponding $\sigma_{v,i}(0)$ values are given in Figure 4 of the main text.
